# The tyrosine kinase inhibitor nilotinib is more efficient than mitotane in decreasing cell viability in spheroids prepared from adrenocortical carcinoma cells

**DOI:** 10.1186/s12935-018-0527-x

**Published:** 2018-03-01

**Authors:** Elaine Silveira, Isadora Pontes Cavalcante, Jean Lucas Kremer, Pedro Omori Ribeiro de Mendonça, Claudimara Ferini Pacicco Lotfi

**Affiliations:** Department of Anatomy, Institute of Biomedical Science, University of São Paulo, São Paulo, SP Brazil

**Keywords:** Adrenocortical carcinoma cells, Nilotinib, Mitotane, Spheroid cell culture

## Abstract

**Background:**

New drugs for adrenocortical carcinoma (ACC) are needed because most patients undergo rapid disease progression despite surgery and adjuvant therapy with mitotane. In this study, we aimed to investigate the in vitro effects of different chemotherapy drugs, alone or combined with mitotane, on the viability of adrenocortical carcinoma cells.

**Methods:**

Everolimus, sunitinib, zoledronic acid, imatinib and nilotinib cytotoxicity, alone or combined with mitotane were tested on ACC H295R cells in monolayer or spheroid cultures using MTS assays and confocal microscopy. Moreover, the nilotinib effects were investigated in spheroids cultured from patient tumor-derived ACC-T36 cells.

**Results:**

Morphological characterization of H295R cell spheroids using histochemistry was performed and showed that dense, homogenously sized, multicellular spheroids were obtained. We observed that sunitinib and nilotinib alone were equally effective in a monolayer preparation, whereas mitotane was the most effective even at a low dose. A combination of sunitinib and mitotane was the most effective treatment, with only 23.8% of cells in the monolayer remaining viable. Spheroid preparations showed resistance to different drugs, although the poor effect produced by mitotane alone was surprising, with a cell viability of 84.6% in comparison with 13.1% in monolayer cells. The most ineffective drugs in spheroid preparations were everolimus, zoledronic acid and imatinib. In both cell types, nilotinib, either alone or in combination with mitotane induced more significant cell viability inhibition in monolayer and spheroid preparations. In addition, the mechanism of nilotinib activity involves the ERK1/2 pathway.

**Conclusion:**

Taken together, our data identified nilotinib as a cytotoxic drug that combined with ERK inhibitors deserves to be tested as a novel therapy for adrenocortical carcinoma.

## Background

Adrenocortical carcinoma (ACC) is a rare malignancy with a dismal prognosis [1]. ACC treatment includes surgery, radiotherapy and adjuvant therapy with mitotane [2]. Despite the treatments utilized, there are no satisfactory therapies for ACC. Different molecular characteristics of ACC could be considered potential therapy targets, including overexpression of vascular endothelial growth factors and receptors (VEGF/VEGFR) and insulin-like growth factors and receptors (IGF/IGFR) [3, 4]; PI3K/AKT/mTOR pathway activation as a mediator of VEGF and IGF [5]; high levels of multidrug resistant protein 1 (MDR-1) [6]; gain of chromosomal region 9q34 [7] and the involvement of ABL oncogene and downregulation of bone morphogenetic proteins 2 and 5 (BMPs) [8].

Everolimus (Eve) acts on mammalian target of rapamycin (mTOR), a key component of the PI3K/Akt pathway, and its antiangiogenic activity and antitumor effects have been reported in adrenocortical tumors [5, 9]. The tyrosine kinase inhibitor sunitinib malate (Sun) potentially inhibits VEGFR1, 2, and 3 and PDGFR-a and PDGFR-b, among others [10], and in adrenocortical carcinoma cells, Sun inhibits cell proliferation and alters steroidogenesis [11]. Nilotinib is a BCR-Abl tyrosine kinase inhibitor and a second-generation form of imatinib, which inhibits the activity of the *ABL* gene family [12] and significantly reverses ABCB1/P-glycoprotein (P-gp) activity in multidrug resistance (MDR) [13]. To our knowledge, nilotinib has not been tested in adrenocortical carcinomas. Zoledronic acid (ZOL) treatment resulted in significant upregulation of BMP-2 gene expression [14], and its antitumor effect was reported in an adrenocortical carcinoma case report [15]. It was previously demonstrated that a combination of mitotane and chemotherapeutic drugs might be more effective in ACC treatment [16]. To determine whether different drugs alone or in combination with mitotane exert antineoplastic activity, we explored their effects on cell viability using H295R cells in monolayer and spheroid preparations. In addition, we analyzed the potential use of tumor cell spheroids from patients to assess their response to chemotherapeutic drugs.

## Methods

### Cell cultures

The NCI-H295R human adrenocortical carcinoma cell line [17] was obtained from the American Type Culture Collection (ATCC, Rockville, MD, USA) and cultured in RPMI supplemented with 2% fetal bovine serum and 1% ITS (all from Gibco, NY, USA). ACC-T36 human adrenocortical carcinoma cells were generated as described in [18] and used between the third and sixth passage. ACC-T36 cells were cultured in DMEM containing 10% FBS (fetal bovine serum), 25 mg/l of ampicillin and 100 mg/l of streptomycin.

### Monolayer cell culture and spheroid preparation

For monolayer culture, 10^4^ cells were plated in triplicate into 96-well plates and maintained at 37° C in a humidified atmosphere containing 95% air and 5% CO_2_. The drug treatments started 24 h after seeding. For spheroid preparation, 10^4^ cells were seeded in triplicate into 96-well plates pre-coated with 1.5% UltraPure™ Agarose (Invitrogen, CA, USA). Cell aggregation was facilitated by plate centrifugation at 1000*g* for 5 min. The plates were incubated at 37° C with 5% CO_2_ for 96 h. Every 2 days, the medium was replenished, and under these conditions, individual multicellular tumor spheroids were generated in each well. After 96 h of incubation, 100 µl of the culture medium was replaced daily with the added drug compounds as indicated in the experiments.

### Histochemistry of spheroid preparations

The spheroids were collected and transferred to glass slides, washed with PBS, and fixed with 3.7% formaldehyde solution (Merck, NJ. USA) for 30 min. For periodic acid–Schiff (PAS) staining, the spheroids were incubated for 5 min in 1% aqueous solution of periodic acid and then with Schiff’s reagent for 15 min, followed by a wash in ammonia water. Collagen staining was achieved using 0.1% Picrosirius red in saturated picric acid for 1 h. After being washed in water, the stained sample was dehydrated, clarified in xylene and mounted in Enthelan (Merck, NJ, USA). Images were captured with a digital microscope camera with and without linear cross polarization. The cells were stained with Oil Red O as described in [19]. Briefly, cells were incubated in freshly prepared Oil Red O working solution for 15 min and rinsed with 50% isopropanol and distilled water. The sections were counterstained with Harry′s hematoxylin for 15 s and mounted in glycerin, and images were captured with a light microscope. To visualize spheroid architecture, spheroids were transferred from wells to a glass-bottomed dish, washed twice with PBS, and fixed with 3.7% paraformaldehyde (Merck, NJ, USA) for 30 min. They were then treated with 0.5% Triton-X 100 followed by staining with Alexa Fluor 568 phalloidin (1:500; Molecular Probes, CA, USA) and 10 μl/ml Hoechst 33342 (Molecular Probes, CA, USA) for 30 min. After being washed with PBS, the spheroids were observed under an inverted confocal microscope (Leica TCS SP8).

### Compounds

Mitotane (1-(2-chlorophenyl)-1-(4-chlorophenyl)-2,2-dichloroethane) and sunitinib malate (*N*-[2-(diethylamino) ethyl]-5-[(Z)-(5-fluoro-1,2-dihydro-2-oxo-3H-indol-3-ylidine) methyl]-2,4-dimethyl-1H-pyrrole-3-carboxamide) were purchased from Sigma (MO, USA). Everolimus (40-*O*-(2-hydroxyethyl)-rapamycin) (RAD001), zoledronic acid (2-(imidazol-1-yl)-hydroxy-ethylidene-1,1-bisphosphonic acid, disodium salt, 4.75 hydrate), nilotinib (AMN107) and Imatinib mesylate (STI571) were kindly provided by Novartis (Basel, Switzerland). Mitotane and everolimus were dissolved in absolute ethanol, sunitinib and nilotinib in dimethyl sulfoxide (DMSO), and zoledronic acid and imatinib in sterile water. Each compound was prepared as a 10 mM stock solution. Experiments were performed with serial dilutions in culture medium from the stock solutions for dilution of solvents. The MEK inhibitor U0126 was purchased from Cell Signaling Technology (MA, USA) and used at a concentration of 10 μM dissolved in DMSO.

Cells were treated with mitotane at 10, 30, and 50 µM; sunitinib at 1.25, 2.5, 5 and 10 µM; and everolimus, zoledronic acid, imatinib and nilotinib at 1, 5 and 10 µM. The concentration utilized considered the therapeutic use range. All drugs were used alone at the concentrations indicated or in combination with low-dose mitotane, 10 or 30 µM, as suggested by [20] for combination with other chemotherapeutic agents. The medium containing the compounds was changed every 24 h for a total of 72 h of treatment.

### MTS assay

After treatment, 20 µl of MTS solution (CellTiter96^®^AQ_ueous_ One Solution Cell Proliferation Assay, Promega, WI, USA) was added, and the plates were incubated at 37 °C for 4 h in a humidified atmosphere of 5% CO_2_. The optical density was read at 490 nm using an ELISA plate reader (BioTek™Epoch™ Microplate Spectrophotometer, Winooski, VT, USA). The spheroids were disaggregated with Accutase^®^ (Gibco, MA, USA) before the addition of MTS solution.

### Confocal microscopy

Viable cells and apoptotic or necrotic cells in spheroids were observed with confocal microscopy (Leica TCS SP8) or an inverted phase contrast microscope (Nikon). Cell labeling with Hoechst, annexin V-FITC and propidium iodide (PI) was performed as described above. The spheroids were transferred to a glass-bottomed dish, and images were obtained using a confocal microscope. Visualization of spheroid images was the same as described above.

### Immunoblotting

For protein analysis, H295R cells were plated at a density of 4 × 10^5^ cells per 35-mm dish. After 48 h, the cells were lysed in ice-cold RIPA buffer (50 mM Tris–HCl, pH 7.5, 150 mM NaCl, 1% NP40, 0.5% sodium deoxycholate, 0.1% sodium dodecyl sulfate) supplemented with protease and phosphatase cocktail (Sigma-Aldrich). Prior to protein extraction, cells were treated with nilotinib alone for 15 or 60 min. In order to investigate the effects of U0126, cells were treated with U0126 for 60 min, and in combined treatment, cells were treated with U0126 for 60 min, followed by treatment with Nilotinib for more 60 min.

The cell pellets were obtained by centrifugation at 14,000 rpm for 15 min at − 4 °C, and total protein was quantified using a Bradford Assay. For each experiment, 15 μg of protein was resolved in 10% SDS-PAGE gels and transferred to nitrocellulose membranes, followed by Ponceau staining to ensure protein transfer. Membranes were blocked using 5% fat-free milk in Tris-buffered saline containing 1% Tween 20 (TBST). Membranes were incubated overnight in TBST containing 5% fat-free milk with the following respective primary antibodies diluted 1:1000: anti-total ERK, anti-phospho ERK, anti-phospho SAPK (all purchased from Cell Signaling Technology, MA, USA) and anti-GAPDH (Santa Cruz Biotechnology, CA, USA). Antibodies were detected with horseradish peroxidase-conjugated secondary antibody and chemiluminescence using ECL reagents in an Imager 600 (GE Healthcare Life Sciences, USA). Quantification of three independent experiments was performed using densitometry with Image J Software.

### Statistical analysis

Statistical analyses were performed with GraphPad^®^ Prism 6.00 software. The results are expressed as the mean ± SD of three different experiments performed in triplicate. Statistical significance (p ≤ 0.05) was determined using two-way ANOVA or paired t test, as indicated.

## Results

### Morphological characterization of spheroids

**Fig. 1:**
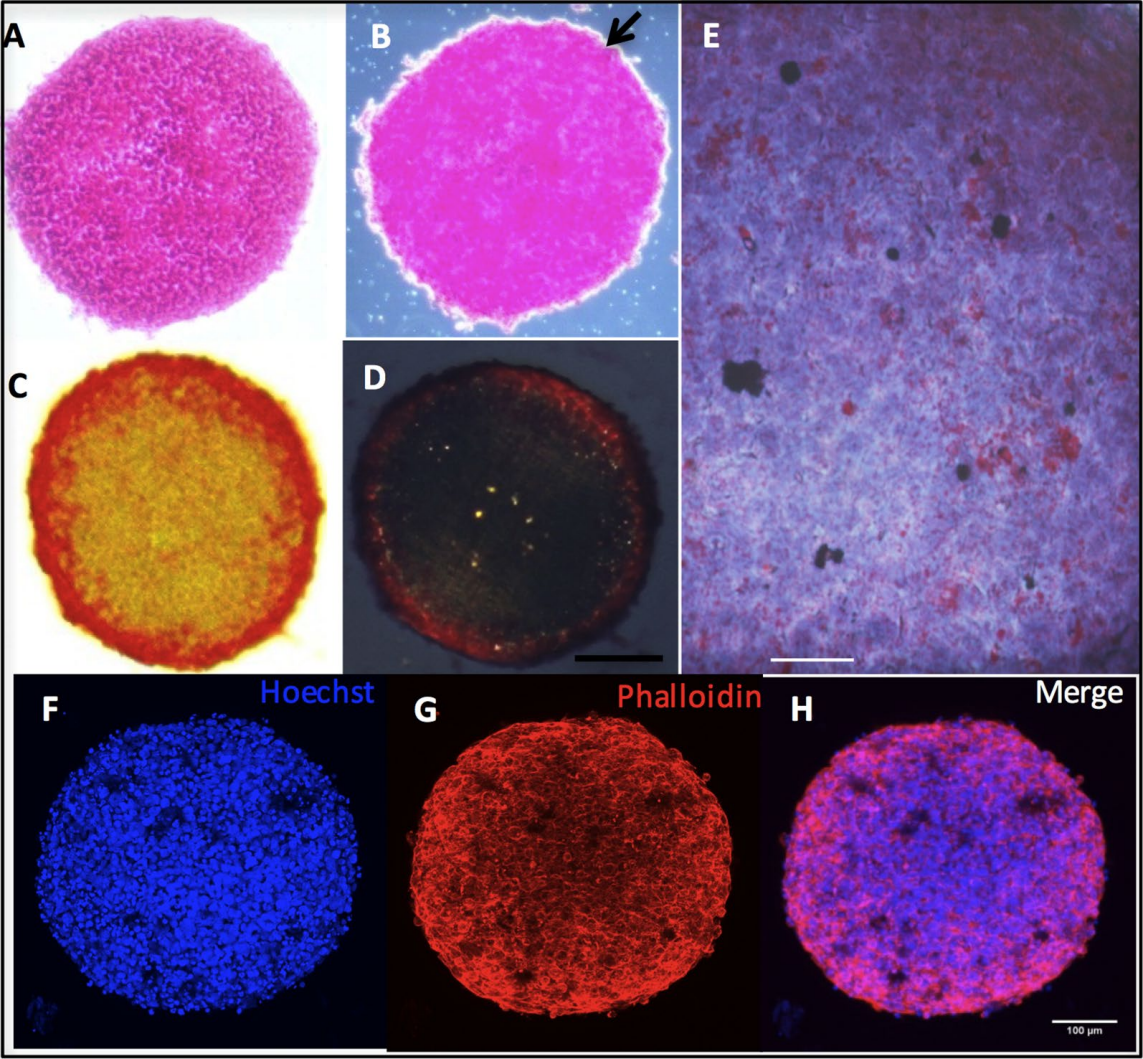
Morphological characterization of H295R cell spheroid preparations. **a** Periodic-acid-Schiff staining of glycoproteins; **b** autofluorescence of collagen fibers; **c** picrosirius red staining visualized with light microscopy; **d** picrosirius red staining of collagen type I (orange/red) and collagen type III (green) imaged with polarized light microscopy; **e** droplets of lipids stained with Oil Red O and **f**–**h** 3D reconstruction of whole-mount confocal Z-stacks of spheroids, stained with Hoechst and phalloidin imaged with confocal microscopy (Leica TCS SP8); 100 μm

**Fig. 2:**
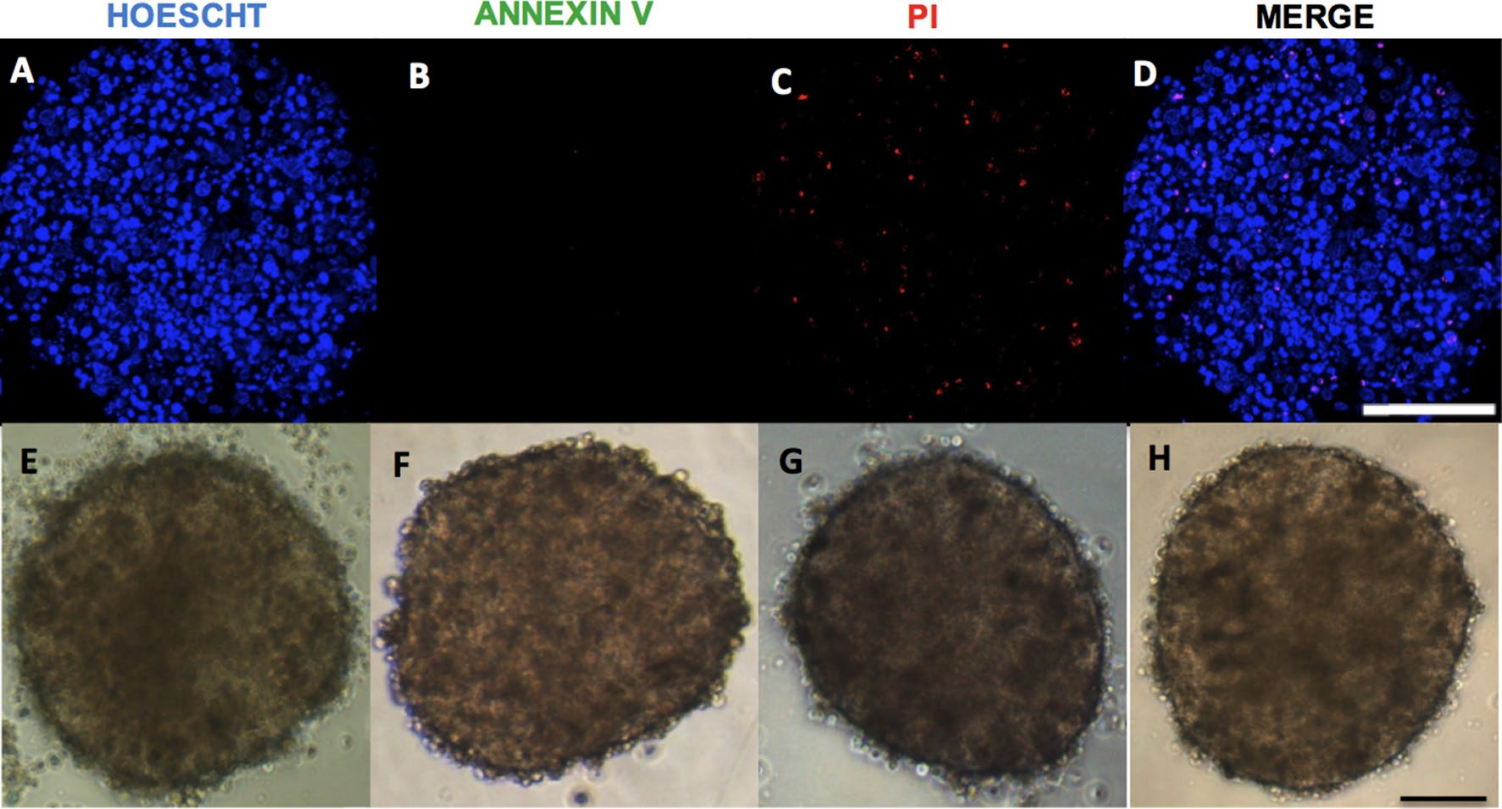
Analysis of viable cells in H295R cell spheroid preparations. **a**-**d** Spheroids were stained with Hoechst (blue), annexin V (green) and propidium iodide (PI) (red) after 96 h of culture. Viable (Hoechst-positive); early apoptotic (annexin V-positive and PI-negative); late apoptotic (annexin V-positive and PI-positive) and necrotic (annexin-negative and PI-positive) cells; **e**–**h** phase contrast photomicrography of spheroids after 24, 48, 72 and 96 h of culture; 100 μm

After 96 h in culture, the spheroids of the H295R cell line produced glycoproteins, revealed by PAS-positive staining (Fig. 1a). The collagen fibers were evidenced by autofluorescence (Fig. 1b) and differentiated with Picrosirius red dye, with collagen type I in orange/red (Fig. 1c) and collagen type III in green (Fig. 1d). Oil Red O-stained lipid droplets were observed in the cytoplasm of cells, a characteristic of steroid-producing cells (Fig. 1e). Reconstruction of spheroids with confocal Z-stacks after Hoechst and phalloidin staining allowed visualization of tight cell–cell interactions (Figs. 1f–h). Analysis of cell viability (Fig. 2) using triple fluorescence staining with Hoechst, annexin V and propidium iodide showed uniform distribution of viable cells after 96 h of culture, no apoptosis and scattered necrotic cells (Fig. 2b–c). Inverted phase-contrast images showed spheroid morphology and their homogeneous size and growth after 24 h until 96 h in culture (Fig. 2e–h) when spheroids were treated as indicated.

### Mitotane treatment exhibited low cytotoxicity in spheroids

**Fig. 3:**
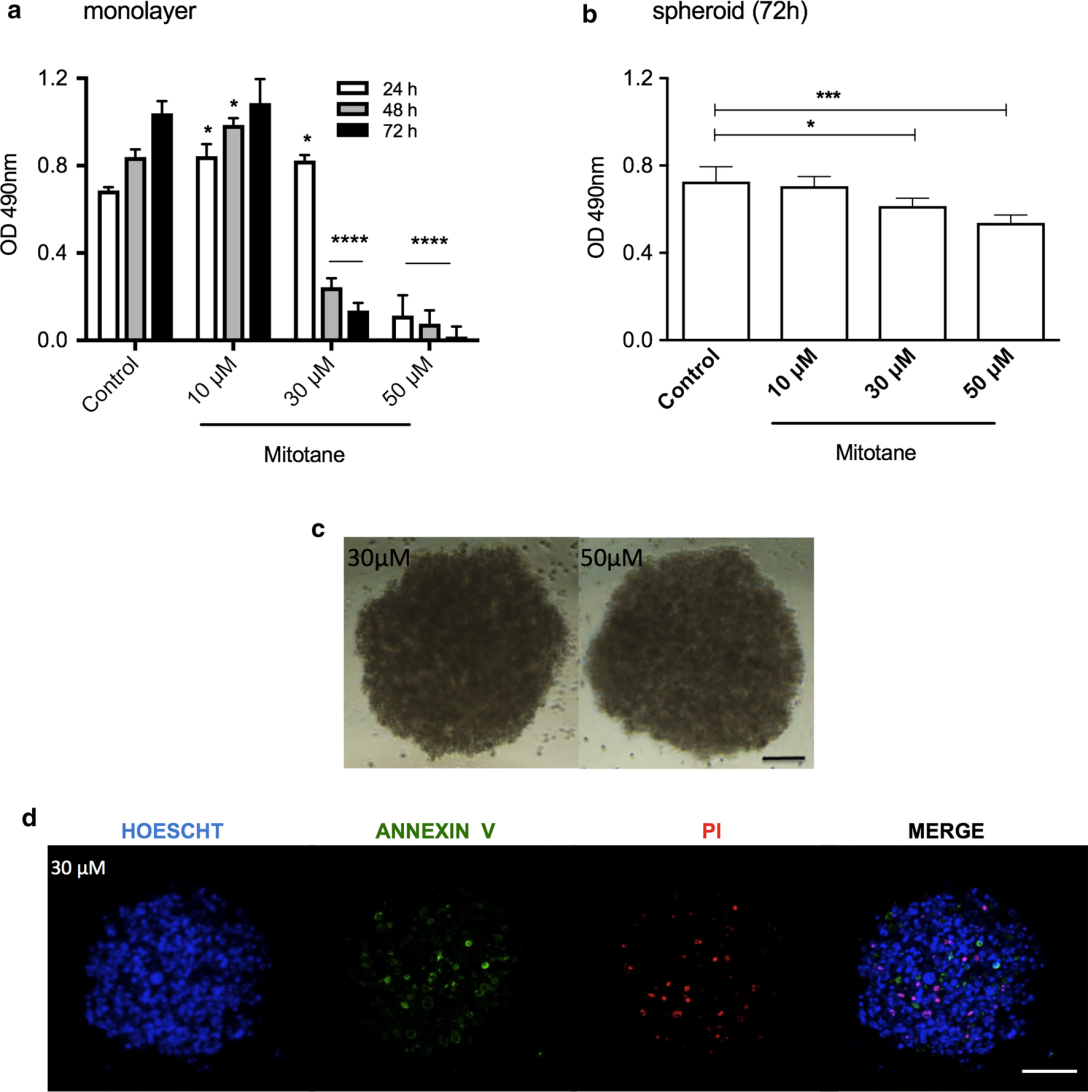
Viability analysis of H295R cells treated with mitotane. **a** MTS assay in monolayer cells after mitotane treatment for 3 days; **b** MTS assay in spheroids after treatment for 72 h; **c** phase-contrast photomicrography of spheroids after 72 h; **d** confocal microscopy images of spheroids after 30 μM mitotane treatment (100 μm). The results are expressed as the mean ± SE; ANOVA *p < 0.05; ***p < 0.001; ****p < 0.0001; n = 3

Three days of treatment with 30 and 50 μM mitotane in the H295R cell line reduced monolayer cell viability by 86.9 and 98.4%, respectively, relative to non-treated cells (Fig. 3a). Conversely, the same treatment in spheroid preparations decreased cell viability by only 15.4 and 26.4%, respectively (Fig. 3b). Consistent with the weak effect of mitotane in spheroids, no modification of their integrity was observed with 30 or 50 μM mitotane (Fig. 3c). Analysis of spheroids treated with 30 μM mitotane using confocal microscopy showed positive labeling for annexin V and PI, indicating a decrease in viability through the induction of, respectively, apoptosis and necrosis (Fig. 3d).

### Nilotinib was the most effective cytotoxic agent in both monolayer and spheroid preparations of adrenocortical tumor cells

**Fig. 4:**
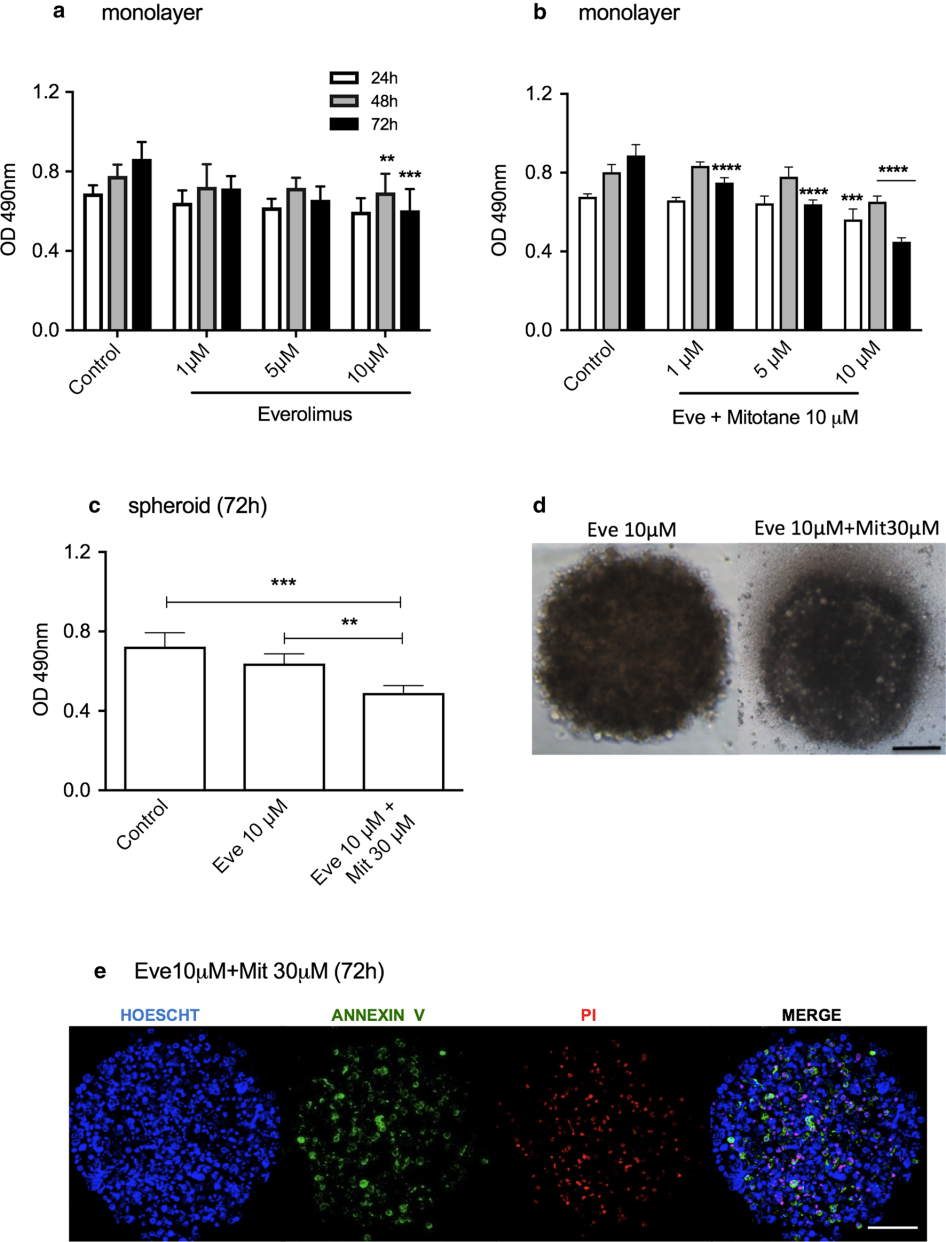
Cell viability analysis of H295R cell line treated with everolimus (Eve) alone or with mitotane (Mit). MTS assay in monolayer cells after everolimus treatment for 3 days (**a**) and (**b**) a combination of Eve and 10 μM Mit for 72 h; (**c**) MTS assay in spheroids after treatment with Eve or a combination of Eve and Mit; **d** phase-contrast photomicrography of spheroids after 72 h of treatment; **e** Confocal microscopy images of spheroids after 72 h of treatment (100 μm). The results are expressed as the mean ± SEM; ANOVA **p < 0.01; ***p < 0.001; ****p < 0.0001; n = 3

Everolimus (10 μM) decreased cell viability by 29.7% after 72 h compared with untreated cells in monolayers (Fig. 4a), and viability was reduced by 49.3% when used in combination with a low concentration of mitotane (10 μM) (Fig. 4b). By contrast, 10 μM everolimus did not present a significant effect in spheroids. A concentration of mitotane three times higher (30 μM) was required to decrease cell viability by 32.1% (Fig. 4c), induce cellular disorganization (Fig. 4d) and increase apoptosis and necrosis (Fig. 4e) after 72 h of treatment.

**Fig. 5:**
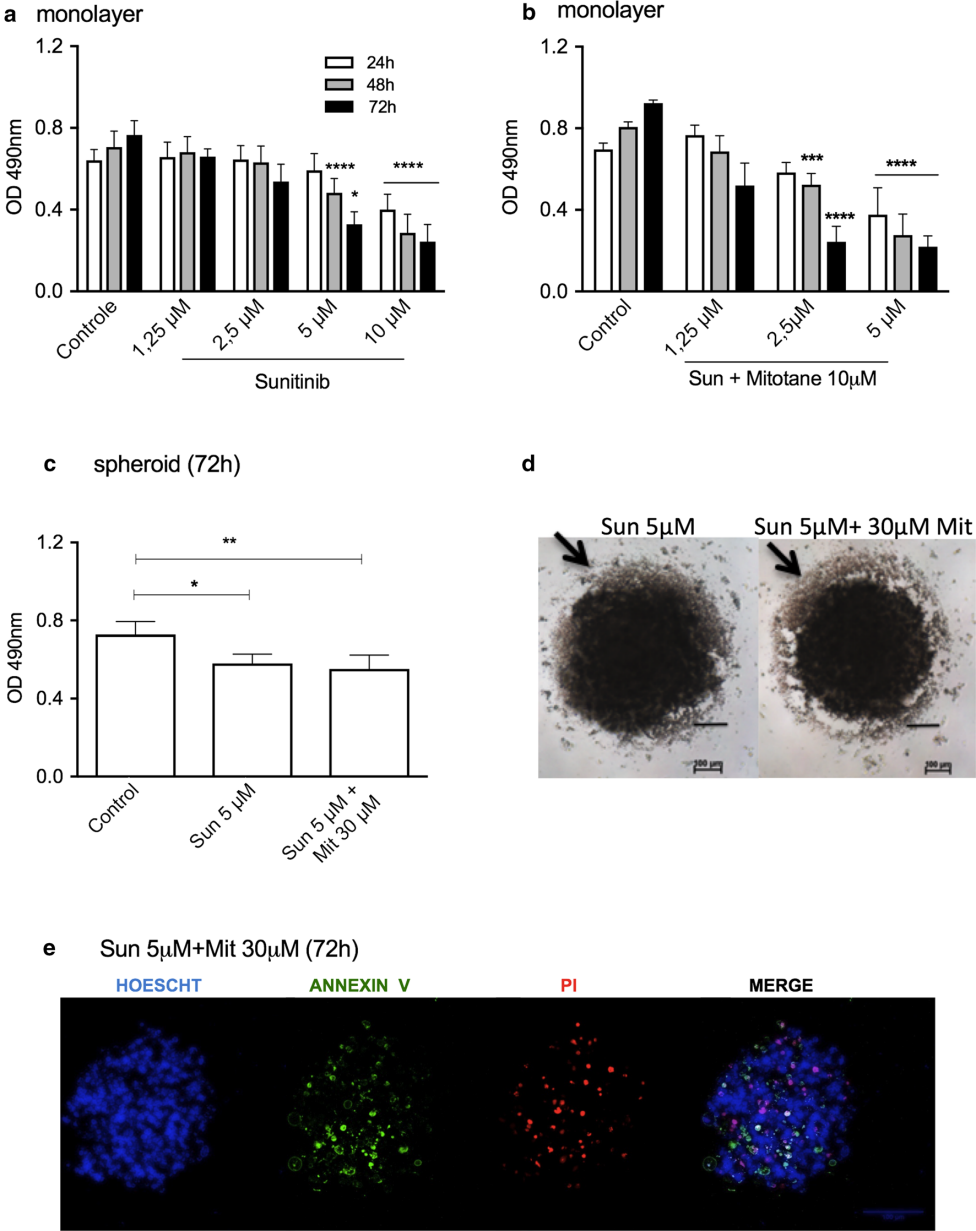
Cell viability analysis of H295R cell line treated with sunitinib (Sun) alone or with mitotane (Mit). MTS assay in monolayer cells after Sun treatment for 3 days (**a**) and (**b**) a combination of Sun and 10 μM Mit for 72 h; (**c**) MTS assay in spheroids after treatment with Sun or a combination of Sun and Mit; (**d**) phase-contrast photomicrography of spheroids after 72 h of treatment; (**e**) confocal microscopy images of spheroids after 72 h of treatment (100 μm). The results are expressed as the mean ± SEM; ANOVA *p < 0.05; **p < 0.01; ***p < 0.001; ****p < 0.0001; n = 3–4

Sunitinib (5 μM) alone or in combination with 10 μM mitotane promoted a decrease in cell viability of 58.4 and 76.2%, respectively, in monolayer cells relative to control cells after 72 h (Fig. 5a, b). In spheroid preparations under the same conditions, we observed a decrease in cell viability of only in 20.3 and 23.6%, respectively (Fig. 5c). However, alone or in combination with 30 μM mitotane, 5 μM Sunitinib promoted strong cell dissociation (Fig. 5d). Confocal images confirmed the presence of viable cells and apoptotic and necrotic cells (Fig. 5e).

**Fig. 6:**
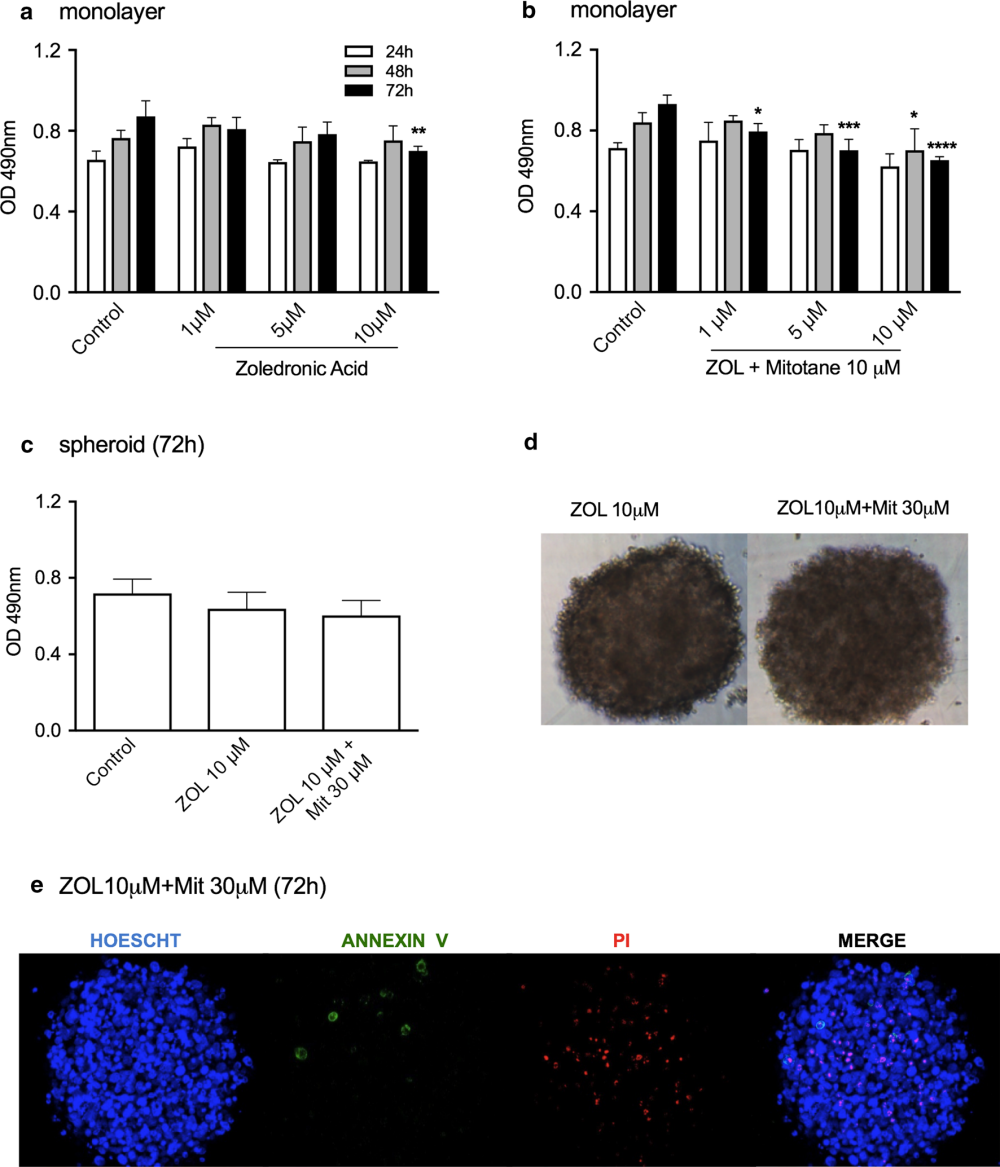
Cell viability analysis of H295R cell line treated with zoledronic acid (ZOL) alone or with mitotane (Mit). MTS assay in monolayer cells after ZOL treatment for 3 days (**a**) and (**b**) a combination of ZOL and 10 μM Mit for 72 h; **c** MTS assay in spheroids after treatment with ZOL or a combination of ZOL and Mit; D) phase-contrast photomicrography of spheroids after 72 h of treatment; **e** confocal microscopy images of spheroids after 72 h of treatment (100 μm). The results are expressed as the mean ± SEM; ANOVA *p < 0.05; ***p < 0.001; ****p < 0.0001; n = 3

In monolayers, 10 μM zoledronic acid (ZOL) reduced the cell viability by 19.5% after 72 h of treatment (Fig. 6a), whereas in combination with 10 μM mitotane, the loss of viability was 30.1% in the same period (Fig. 6b). No significant difference was observed in spheroids treated with ZOL alone or in combination with mitotane (Fig. 6c). While the integrity of spheroids was barely modified (Fig. 6d), confocal microscopy images confirmed the weak effect of ZOL in spheroid preparations (Fig. 6e).

**Fig. 7:**
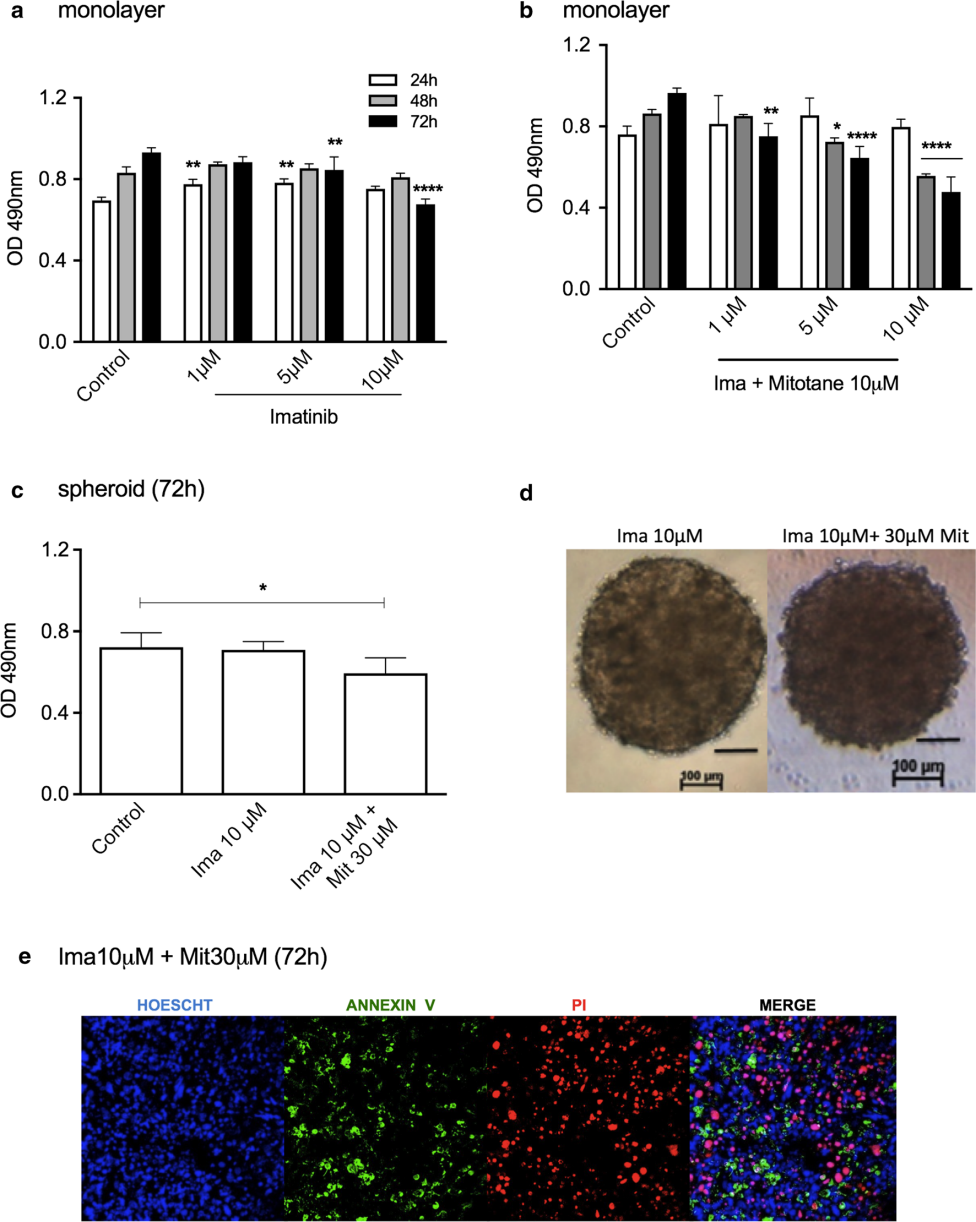
Cell viability analysis of H295R cell line treated with imatinib (Ima) alone or with mitotane (Mit). MTS assay in monolayer cells after Ima treatment for 3 days (**a**) and (**b**) a combination of Ima and 10 μM Mit for 72 h; **c** MTS assay in spheroids after treatment with Ima or a combination of Ima and Mit; **d** phase-contrast photomicrography of spheroids after 72 h of treatment; **e** Confocal microscopy images of spheroids after 72 h of treatment (100 μm). The results are expressed as the mean ± SEM; ANOVA *p < 0.05; **p < 0.01; ***p < 0.001; ****p < 0.0001; n = 3-4

Treatment with 10 μM imatinib decreased the monolayer cell viability by 27.2% relative to control cells after 72 h of treatment (Fig. 7a). In combination with 10 μM mitotane, it was more effective, diminishing the cell viability by 50.3% in the same period (Fig. 7b). While the effect of imatinib alone was weak in monolayers, it was ineffective in spheroids. Together with 30 μM mitotane, the reduction in viability was 18.1% (Fig. 7c), which does not promote modification of spheroid morphology (Fig. 7d). Confocal microscopy images revealed apoptosis and necrosis in spheroid preparations treated with 10 μM Imatinib and 30 μM mitotane (Fig. 7e).

**Fig. 8:**
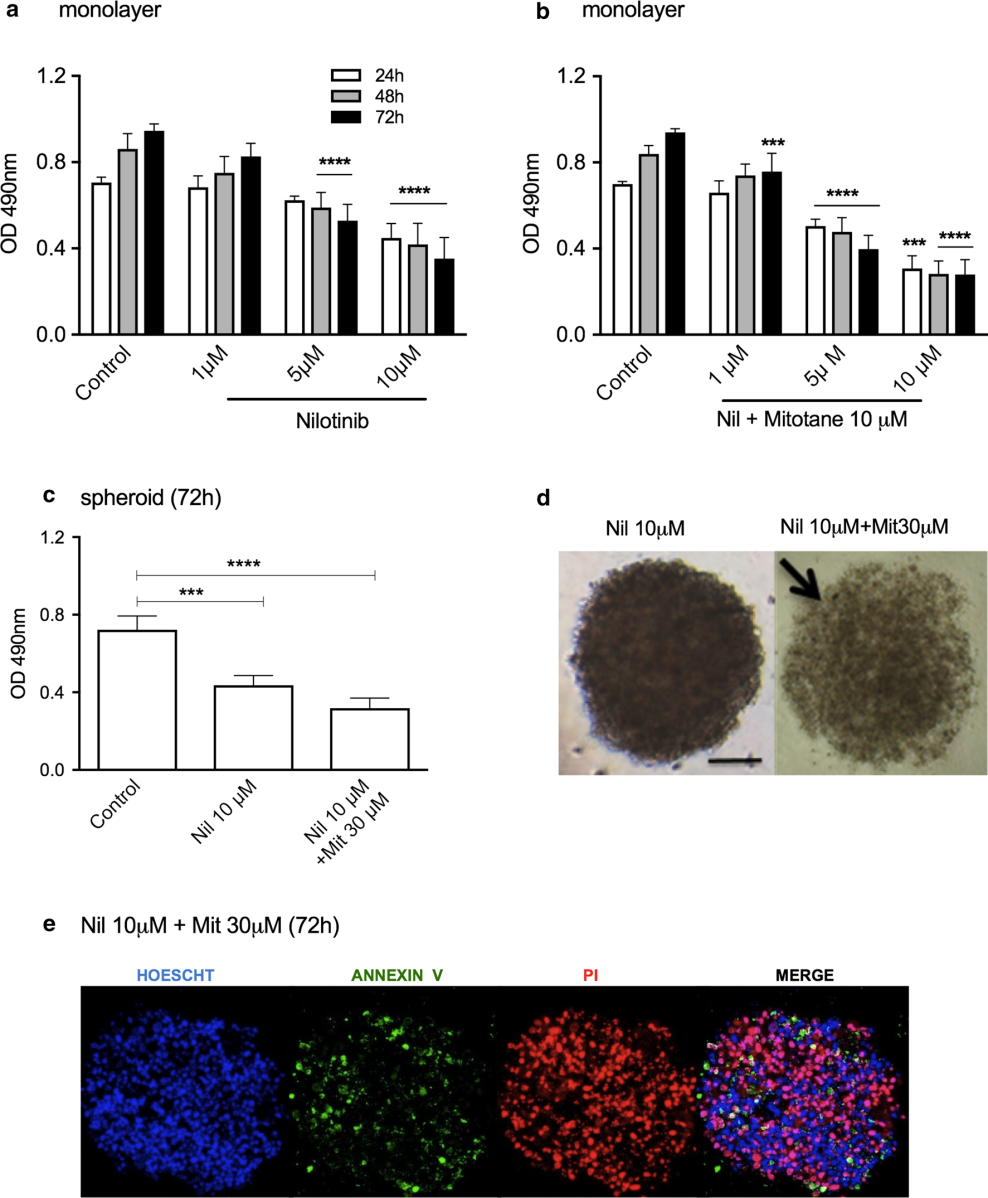
Cell viability analysis of H295R cell line treated with nilotinib (Nil) alone or with mitotane (Mit). MTS assay in monolayer cells after Nil treatment for 3 days **a** and **b** a combination of Nil and 10 μM Mit for 72 h; **c** MTS assay in spheroids after treatment with Nil or a combination of Nil and Mit; **d** phase-contrast photomicrography of spheroids after 72 h of treatment; **e** Confocal microscopy images of spheroids after 72 h of treatment (100 μm). The results are expressed as the mean ± SEM; ANOVA ***p < 0.001; ****p < 0.0001. The results are expressed as the mean ± SEM; n = 3–4

Nilotinib (10 μM) alone decreased cell viability in monolayer culture by 62.9% (Fig. 8a), whereas in combination with 10 μM mitotane the reduction reached 70.2% after 72 h of treatment (Fig. 8b). In spheroid preparations, the reduction of viability with 10 μM nilotinib was 39.6%, whereas to reach a cell viability reduction of 56.0%, it was necessary to combine 10 μM nilotinib and 30 μM mitotane (Fig. 8c). This drug combination induced spheroid disaggregation (Fig. 8d). In addition, confocal microscopy images of spheroids showed high levels of apoptosis and necrosis, suggesting high cytotoxicity levels after treatment with a combination of 10 μM nilotinib and 30 μM mitotane (Fig. 8e).

**Fig. 9:**
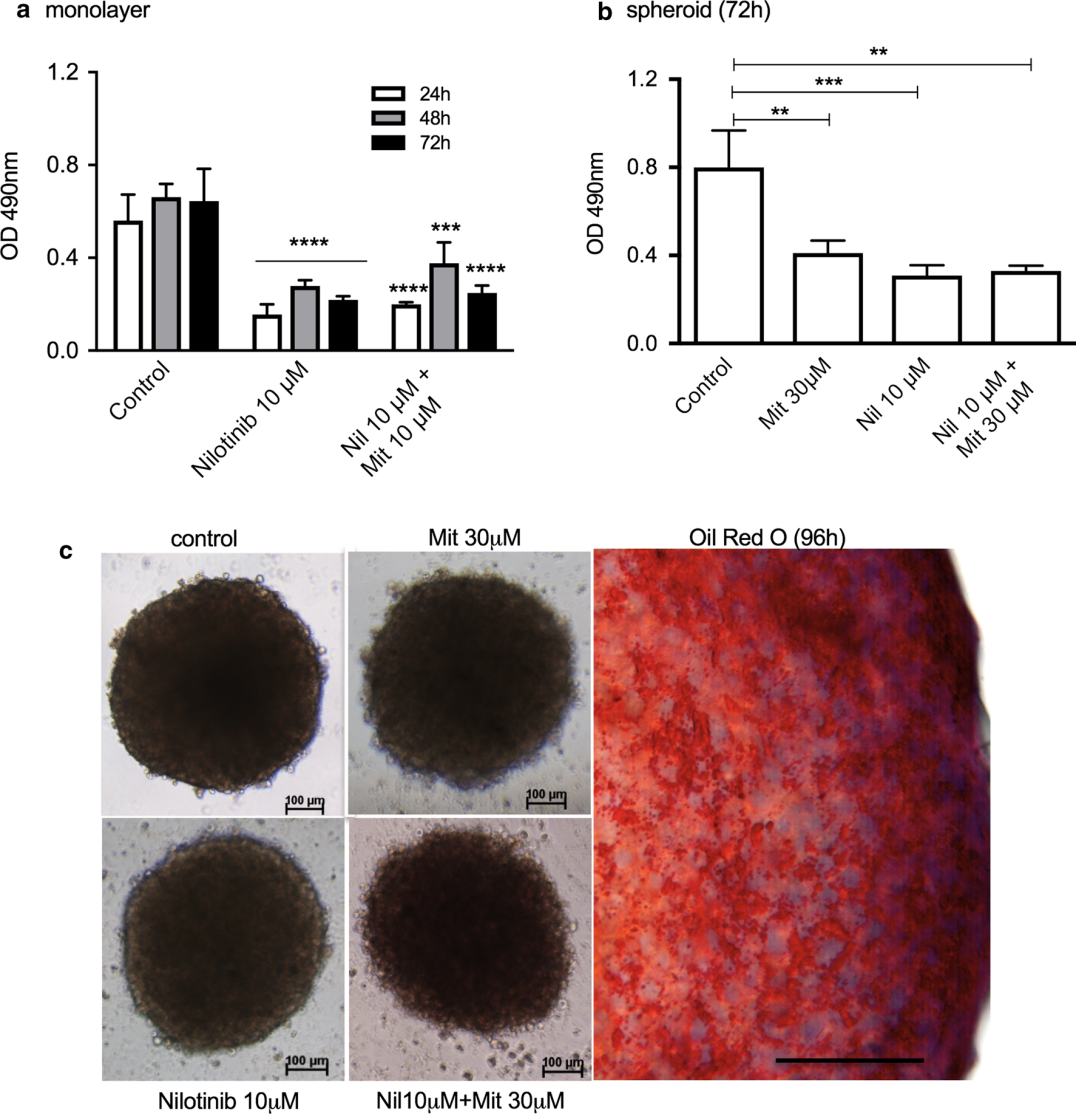
Cell viability analysis of ACC-T36 cell cultures treated with nilotinib (Nil) alone or with mitotane (Mit). MTS assay after Nil treatment for 3 days or a combination of Nil and 10 μM Mit in monolayer cells (**a**) and (**b**) in spheroid preparations; **c** phase contrast photomicrography of spheroids treated for 72 h and control cells (100 μm) stained with Oil Red O. The results are expressed as the mean ± SEM; ANOVA **p < 0.01; ***p < 0.001; ****p < 0.0001; n = 3

To validate the effect of Nilotinib in a culture of adrenocortical cells obtained from a patient, patient-derived adrenocortical carcinoma ACC-T36 cells were cultured [18]. An MTS assay detected a reduction in viability of 67.2% with 10 μM nilotinib alone, which was not potentiated in combination with 10 μM mitotane, which showed a decrease in viability of 62.5% in monolayer cells (Fig. 9a). Similarly, spheroid preparations showed a reduction in viability of 62 and 60.0% after treatment with 10 μM nilotinib alone and in combination with 30 μM mitotane, respectively (Fig. 9b). These results of nilotinib in the spheroid preparation contrast with the reduction in cell viability of 15.4% observed in the H295 cell line (Fig. 3c). In Fig. 9c, phase contrast images of ACC-T36 spheroids after different treatments can be observed, and cytoplasmic lipid droplets stained with Oil Red O, which characterize steroidogenic cells, are present. Additionally, in Fig. 9c, spheroid disaggregation after treatment with a combination of 10 μM nilotinib and 30 μM mitotane is evident. All the above results are summarized in Table 1.

**Table 1:** Comparative analysis of cell death (%) of monolayer and spheroid ACC cells analyzed by MTS assay

	Monolayer		Spheroids	
Treatment^a^				
Mitotane	10 μM	30 μM	10 μM	30 μM
	0	86.9	0	15.4
Mitotane^b^				48.1

	+ Mitotane (10 μM)		+ Mitotane (30 μM)	
Everolimus (10 μM)	29.7	49.3	0	32.1
Sunitinib (5 μM)	58.2	76.2	20.3	23.6
Zol Acid (10 μM)	19.5	30.1	0	0
Imatinib (10 μM)	27.2	50.3	0	18.1
Nilotinib (10 μM)	62.8	70.2	39.6	56.0
Nilotinib^b^ (10 μM)	67.2	62.5	62.0	60.0

*ACC* adrenocortical carcinoma

^a^For 72 h

^b^ACC-T36

### The mechanism of nilotinib involves the ERK1/2 pathway

**Fig. 10:**
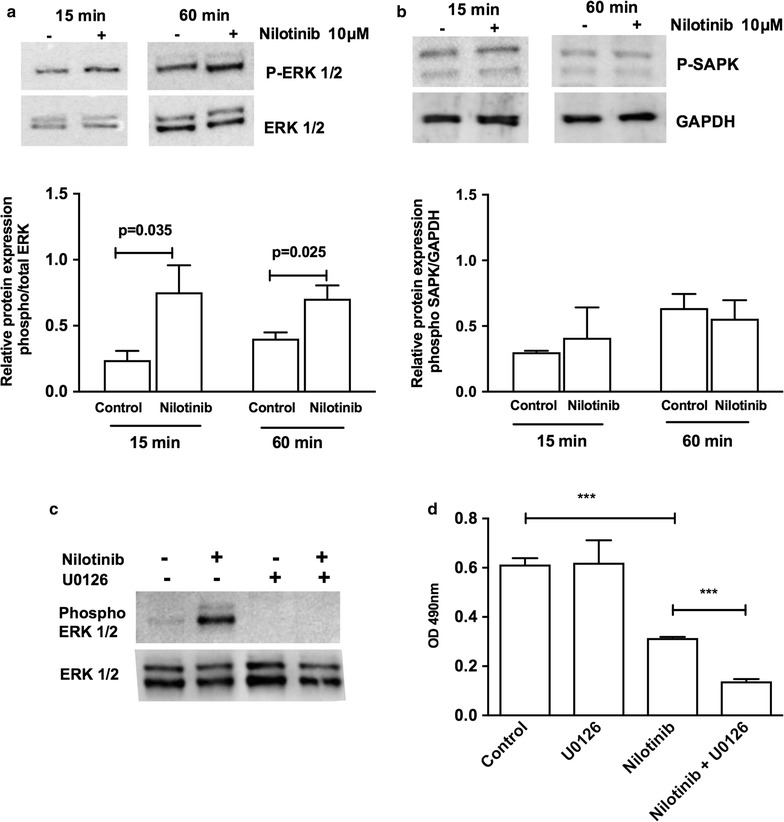
Relative protein expression of phosphorylated and total ERK1/2 (**a**) and phosphorylated SAPK (**b**) in H295R cells. (−) treated with DMSO or (+) 10 μM Nilotinib for 15 and 60 min. The results are expressed as the mean ± SD. Paired t test, n = 3. Relative protein expression of phosphorylated and total ERK1/2 in H295R cells (−) treated with DMSO or (+) 10 μM U0126 for 60 min or/and (+) Nilotinib for 60 min (**c**). Cell viability analysis via MTS assay after 48 h (**d**); the results are expressed as the mean ± SD. ANOVA *** p < 0.001; n = 3

Given that ERK1/2 is known to influence tyrosine kinase inhibitor resistance in tumor cells [42, 43], we investigated the activation of the ERK pathway in the presence of nilotinib. We observed activation of ERK1/2 phosphorylation in monolayer H295R cells treated with nilotinib for 15 and 60 min (Fig. 10a). In contrast, we did not observe activation of SAPK/JNK in H295R cells (Fig. 10b). To determine if ERK activation could interfere with the action of nilotinib, we treated the monolayer H295R cells with both nilotinib and the MEK inhibitor U0126. We observed that after treating H295R cells with both nilotinib and U0126, ERK1/2 phosphorylation was efficiently inhibited (Fig. 10c), and through MTS assays, the decrease in cell viability was greater than with nilotinib alone (Fig. 10d).

## Discussion

There is evidence that cancer cells growing in vitro as spheroids more accurately mimic the drug sensitivity and resistance found in vivo than under monolayer conditions [21]. Spheroid cell culture can modify cell shape, cell–cell and cell–matrix interactions and gene expression, and cells display different growth rates compared to monolayer cultured cells [22]. In addition, spheroids simulate important tumor characteristics in vivo, such as hypoxia, dormancy, and anti-apoptotic features, which result in drug resistance [23].

The method of preparing spheroids from adrenocortical carcinoma cells showed that 96 h was an appropriate time for preparation of single, homogenously sized, dense multicellular spheroids without necrosis. In addition, we also observed cell–cell interactions and production of extracellular matrix and basal membrane proteins in the H295R cell spheroid preparations. The detection of collagen type I and III also confirms the presence of reticular fibers, which are abundant in the adrenal cortex [24]. In addition, the presence of actin, which is responsible for the densely packed organization of the cells, can influence drug diffusion and promote a drug-resistant microenvironment [25].

Despite the side effects, mitotane has been clinically used for decades as adjuvant therapy for adrenocortical carcinoma. As observed here and described by others [26, 27], in monolayer culture only 1.6% of cells remain viable after treatment with 50 μM mitotane, which corresponds to the therapeutic dose (14–20 mg/ml or 44–62 μM), whereas 13.1% viability remains with 30 μM treatment. The spheroid preparations showed resistance to the cytotoxic effect of 30 μM mitotane, maintaining cell viability of 84.6%, although in ACC-T36 spheroids, this concentration was more effective (cell viability of 60%). The weak effect of mitotane in spheroids can also be visualized in confocal microscopy images, in which few apoptotic or necrotic cells were observed after 72 h of treatment.

In general, studies using anticancer agents have shown that tumor cells are less sensitive to therapeutic agents when analyzed in spheroids compared with monolayer conditions [22]. That difference occurs due to hypoxia, inhibition of apoptosis and the low permeability of spheroids [21]. Conversely, activation of human epithelial growth factor (HER2) homodimers formed in spheroid preparations of breast cancer cells resulted in enhanced inhibition of proliferation in cancer cells treated with trastuzumab (Herceptin), a monoclonal antibody specifically targeting HER2 [28]. Irrespective of sensitivity in spheroids and monolayers, spheroids potentially provide a more accurate representation of avascular tumors in vivo. Because of easy handling and the possibility of preparation of spheroids with mosaics of different cells, such as fibroblasts, endothelial cells, and immune cells, spheroid preparations are a powerful tool for drug screening using cell cultures from patient tumors.

Several studies have shown the involvement of IGF1/IGF1R and mTOR pathways in the pathogenesis of adrenocortical carcinoma. Inhibitors of both pathways alone and in combination decreases cell proliferation in vitro and in tumor xenografts in vivo [4, 5, 29, 30]. Using everolimus, an inhibitor of mammalian target of rapamycin, in tumors of different histological types, we observed a weak effect of everolimus alone and a moderate effect in combination with mitotane. However, in spheroid preparations, everolimus did not present an effect and in combination with mitotane caused a poor cytotoxic effect. The results in spheroids agree with a study of everolimus therapy for progressive adrenocortical tumors, in which no clinically meaningful response was observed with everolimus in four patients with advanced ACC [31]. Moreover, a recent study of a combination of everolimus and mitotane in vitro suggests caution in designing combinations of mitotane with other drugs such as mTOR inhibitors or somatostatin analogs [32].

Sunitinib has been successfully used in metastatic renal cell carcinoma and gastrointestinal stromal tumors [33, 34]. It was described as effective according to a case report of a VEGF-positive patient presenting with metastatic ACC [35]. In another study [36], sunitinib serum levels of patients were reduced by mitotane-induced P450-3A4 activity, attenuating its activity. Despite these negative interactions, our results showed poor and similar cytotoxic effects of sunitinib alone or in combination with mitotane in spheroid preparations, although in H295R monolayer cells, it was the most effective cytotoxic drug.

Imatinib and nilotinib are tyrosine kinase inhibitors (TKIs) that are the first-line treatments for chronic myeloid leukemia (CML) [37]. An efficacy and safety study of imatinib in patients with endocrine tumors that expressed c-kit and/or PDGF-R showed no objective responses in 15 patients with disseminated endocrine tumors, including adrenocortical carcinoma [38]. In agreement with the results observed in patients, the effect of imatinib alone was weak in monolayer H295R cells and ineffective in spheroids.

Nilotinib is a second generation TKI that functions via ATP-competitive inhibition. In addition to Bcr-Abl inactivation, it also inhibits a broad spectrum of kinase-suppression activity kinases [39]. Nilotinib also suppressed proliferation in the estrogen-deprived breast cancer cell line MCF-7 [40], and metastatic melanoma cells expressing c-Abl/Arg kinase activity are also susceptible to nilotinib-mediated cell growth inhibition [41]. Our present data show that nilotinib was efficiently cytotoxic in spheroid preparations of both the adrenocortical carcinoma cell cultures utilized and was more efficient than imatinib.

In some types of tumors, ERK1/2 activation can promote tyrosine kinase inhibitor (TKI) resistance [42, 43], influencing treatment efficacy. In this study, we described in adrenocortical carcinoma cells a robust nilotinib capacity to decrease cell viability, despite higher ERK activation compared to control cells. This mechanism was observed in prostate cancer cells [44], and it is not yet known whether the increase in ERK phosphorylation is an abnormal mechanism of resistance or if nilotinib treatment induces a selection of ERK-positive cells [45]. Moreover, sustained and acute activation of SAPK/JNK can be involved in apoptosis and cell survival, respectively [46, 47]. In ovarian cancer cells, it was observed that Nilotinib decreased cell viability, despite SAPK/JNK activation [48]; however, in this study, we did not observe a significant difference in this pathway after nilotinib treatment. Taken together, these results suggest that nilotinib treatment can be influenced by ERK activation but not by SAPK/JNK activation in adrenal cancer cells. Finally, the combination of nilotinib with a MEK inhibitor enhances the cytotoxicity of this compound in H295R cells, which can be an advantage in adrenal cancer therapy.

## Conclusions

In this study, we demonstrated that adrenocortical tumor cells formed dense multicellular spheroids and that the formation of spheroids was associated with decreased sensitivity to chemotherapy drugs. Spheroids of cultured adrenocortical cells showed resistance to different drugs, primarily to mitotane, which is by far the most widely used drug in the treatment of adrenocortical carcinoma. In addition, we demonstrated that the cytotoxic effect of nilotinib is equally efficient in both monolayer and spheroid preparations of adrenocortical carcinoma cells derived from a patient, independent of the presence of mitotane. Taken together, our data identified nilotinib as an important compound that can be used in the treatment of adrenocortical carcinoma and suggested that combined therapy with ERK inhibitors could be a novel therapy for this type of tumor.
